# Metal-Organic Decomposition-Mediated Nanoparticulate Vanadium Oxide Hole Transporting Buffer Layer for Polymer Bulk-Heterojunction Solar Cells

**DOI:** 10.3390/polym12081791

**Published:** 2020-08-10

**Authors:** Chengkai Xia, Won Tae Hong, Young Eun Kim, Woo-Seok Choe, Dong-Hwan Kim, Jung Kyu Kim

**Affiliations:** School of Chemical Engineering, Sungkyunkwan University (SKKU), Suwon 16419, Korea; xckielts@gmail.com (C.X.); wontae788@gmail.com (W.T.H.); yeunn1288@gmail.com (Y.E.K.); checws@skku.edu (W.-S.C.)

**Keywords:** V_2_O_5_, hole transport layer, organic solar cells, low-temperature solution-process, stability

## Abstract

In this study, a solution-processable compact vanadium oxide (V_2_O_5_) film with a globular nanoparticulate structure is introduced to the hole transport layer (HTL) of polymer bulk-heterojunction based solar cells comprised of PTB7:PC_70_BM by using a facile metal-organic decomposition method to replace the conventionally utilized poly(3,4-ethylenedioxythiophene): poly(styrenesulfonate) (PEDOT:PSS). For this, a biocompatible structure-determining agent, polyethylene glycol (PEG, *M_n_* 300), is used as an additive in the precursor to form the nanoparticulate compact V_2_O_5_ (hereafter referred to as NP-V_2_O_5_) film, which possesses an outstandingly smooth surface morphology. The introduction of NP-V_2_O_5_ HTL via the solution process with a neutral pH condition successfully improved the stability by preventing the decomposition of indium tin oxide (ITO) glass and the penetration of heavy-metal components and moisture, which are considered as the crucial drawbacks of using PEDOT:PSS. Over 1440 h (60 days) of the stability test, an organic solar cell (OSC) with NP-V_2_O_5_ showed a significant durability, maintaining 82% of its initial power conversion efficiency (PCE), whereas an OSC with PEDOT:PSS maintained 51% of its initial PCE. Furthermore, due to the positive effects of the modified surface properties of NP-V_2_O_5_, the PCE was slightly enhanced from 7.47% to 7.89% with a significant improvement in the short-circuit current density and fill factor.

## 1. Introduction

Organic solar cells (OSCs) with a donor–acceptor bulk-heterojunction (BHJ)-based active layer composed of semiconductive conjugated polymers and electron acceptors have attracted considerable attention due to their cost-effective solution-based processability [1,2,3]. The power conversion efficiency (PCE) of OSCs strongly depends on the extraction performance of the separated charge carriers from the BHJ active layer to each charge transporting layer and charge collection layer [4]. For obtaining high-efficiency single-junction OSCs, balancing the collection of photo-induced electrons and holes from the active layer to each charge collector is considered a critical property considering the device structure design [5,6,7]. In the state-of-the-art BHJ OSCs with the conventional structure, poly(3,4-ethylenedioxythiophene):poly(styrenesulfonate) (PEDOT:PSS) has been extensively utilized as a hole transporting material because of its unique properties, such as forming a surface dipole with a thickness less than 50 nm and extracting holes from the active layer to the indium tin oxide (ITO) glass substrate with an appropriate work-function of approximately 5.2 eV vs vacuum [8,9,10]. In addition, the PEDOT:PSS layer can ameliorate the contact property of the active layer against the surface state of the anodic substrate, which encourages the use of PEDOT:PSS as a buffer layer between the active layer and the anode [11,12]. Thus, commercialized PEDOT:PSS such as Clevios P VP AI 4083 has been extensively utilized as the hole transport layer (HTL) for achieving high-efficiency OSCs in recent years [5,13,14,15]. However, the deposition of a PEDOT:PSS solution on ITO glass deteriorates the device performance and durability, as its strong acidic condition with pH ~2 can dissolve the metal components, including indium element [11]. Especially, during the spin-coating process of a PEDOT:PSS layer, the sulfonate groups in the PSS dopant can accelerate the dissolution of heavy metal components in ITO [16,17,18,19]. The dissolved indium element from ITO can penetrate the active layers, resulting in poor device stability during the device operation [8,11,20]. Thus, the development of a novel HTL having a high durability and efficient hole migration property with a high selectivity is critical for the replacement of the PEDOT:PSS layer in OSCs.

Recently, a vanadium oxide (V_2_O_5_) thin film has been considered a promising HTL for diverse solar cells, owing to its appropriate work-function of 4.7 eV with the band gap of 2.4 eV [21,22,23,24]. Moreover, a crystalline V_2_O_5_ film can be obtained at a relatively lower temperature compared with other metal oxides (over ~300 °C) [25]. In addition, a V_2_O_5_ HTL can provide a much higher hole selectivity than other p-type metal-oxide-based HTLs, including molybdenum oxide (MoO_3_), resulting in an enhanced open-circuit voltage (*V_oc_*) [26,27]. However, solution-processed crystalline V_2_O_5_ films suffer from a high surface roughness and large porosity, which diminish the device performance [23,28]. Consequently, alternative approaches for introducing PEDOT:PSS to V_2_O_5_ to form a double-decked HTL have been proposed in recent years, to alleviate these drawbacks [24,29,30]. This strategy can enhance the PCE of solar cells. However, challenges remain in replacing PEDOT:PSS and achieving a high durability of the device.

In this study, a solution-processable compact V_2_O_5_ film with a nanoparticulate structure was introduced to the hole transporting buffer layer between the active layer comprised of PTB7:PC_70_BM and an ITO glass substrate by using a facile metal-organic decomposition method to replace PEDOT:PSS. Accordingly, as a biocompatible structure-determining agent, polyethylene glycol (PEG, *M_n_* 300) was utilized to fabricate the nanoparticulate compact V_2_O_5_ (hereafter referred to as NP-V_2_O_5_) film, which possesses an outstandingly smooth surface morphology. PEG 300 has been extensively used as an additive to fabricate globular nanoparticulate films via various solution-based processes due to its good affinity with transition metal precursors and low thermal decomposition temperature (less than 300 °C) [31,32]. Furthermore, due to its low-temperature solution process with an annealing temperature of approximately 400 °C, the ITO glass substrate remains intact [33]. Consequently, the introduction of NP-V_2_O_5_ to the HTL via the solution process with a neutral pH condition successfully improved the stability of OSCs by preventing the decomposition of ITO glass. A stability test was conducted over 1440 h (60 days) to investigate the durability of OSCs. An OSC with NP-V_2_O_5_ showed high durability, maintaining 82% of its initial PCE, whereas an OSC with PEDOT:PSS maintained 51% of its initial PCE. Furthermore, due to the positive effects of the modified surface energy and surface properties of NP-V_2_O_5_, the PCE was slightly enhanced from 7.27% to 7.89% with a significant improvement in the short-circuit current density (*J_sc_*) and fill factor (*FF*).

## 2. Materials and Methods

### 2.1. Fabrication of Devices

The facile metal-organic decomposition process was performed to fabricate the nanoparticulated V_2_O_5_ buffer layer. A precursor solution of concentration 100 mM was prepared by dissolving vanadium acetylacetonate (Sigma-Aldrich, Incheon, Korea) in acetylacetone (Sigma-Aldrich, Korea), and its pH was adjusted using an NH_4_OH solution (Samchun Chem, Seoul, Korea). After transferring the precursor solution to a heating mantle with an Ar-filled flask at 80 °C, polyethylene glycol (Sigma-Aldrich, Korea) of various concentrations and 50 mM α-terpineol (Sigma-Aldrich, Korea), were added to the precursor solution and stirred for 1 day under a moderate stirring of 300 rpm. The solution was filtered by using a cellulose syringe filter with the porosity of 200 nm, and subsequently spin-cast onto the surface of a pre-cleaned ITO glass substrate and calcined at 400 °C for 2 h. UV-ozone treatment was conducted for 20 min on the pre-cleaned ITO substrate before spin-casting the solution, to improve the surface energy of the ITO glass. The thickness of nanoparticulated V_2_O_5_ layer with optimal condition was approximately 80 nm. The active layer with a thickness of approximately 100 nm was spin-coated on the surface of the vanadium oxide layer in a globe box filled with Ar gas. The BHJ solution was prepared by mixing poly((4,8-bis[(2-ethylhexyl)- oxy]benzo[1,2-b:4,5-b′]dithiophene-2,6-diyl)(3-fluoro-2-[(2-ethylhexyl)carbonyl]thieno[3,4-b]thiophenediyl)) (PTB7, 1-Material) and the fullerene derivative [6,6]-phenyl-C71-butyric acid methyl ester (PC_70_BM, Nano-C) in a weight ratio of 1:1.5 to fabricate a 2.5 wt% solution, where the solvent was prepared by mixing 1,8-diiodooctane (DIO, Sigma-Aldrich, Korea) in chlorobenzene (3 v%). After drying the BHJ layer in a vacuum chamber (approximately 10^−2^ Torr, Daedong High Tech., Seoul, Korea) for 24 h, a 20 mM titanium oxide layer with the thickness of approximately 6 nm was spin-coated as an electron transport layer (ETL). Then, the sample was moved to a thermal evaporator and a 100-nm-thick Al metal cathode was deposited (lower than 10^−6^ Torr, Daedong High Tech., Korea). The post-thermal treatment was conducted at 85 °C in the Ar-filled glove box.

### 2.2. Characterization

The surface properties of the vanadium oxide and BHJ layers were characterized using field-emission scanning electron microscopy (SEM, JSM-7001F, JEOL Ltd., Tokyo, Japan), atomic force microscopy (AFM, XE-100, Park Systems, Suwon, Korea) in tapping mode, and optical contact-angle measurement (Mirae S Tech Co., Ltd., Bucheon, Korea). X-ray diffraction (XRD, D8 Advance X-ray diffractometer, Bruker, MA, USA) measurements were performed with an operating voltage of 40 eV. X-ray photoelectron spectroscopy (XPS) was conducted using a VG K-alpha system (Thermo, Waltham, MA, USA) whose binding energies were calibrated using the C-C bond in the C 1s peak centered at 284.8 eV. For the characterization of the device performance, a model 2400 source meter (Keithley, MA, USA) under a simulated AM 1.5G 1 sun illumination using a solar simulator (PEC-L01, Peccell Technology, Tokyo, Japan) was utilized to obtain the *J–V* curves. The intensity of the solar simulator (100 mW/cm^2^) was calibrated using a Si reference cell (P/N 91150V, VLSI standards, Stratford, CT, USA). The external quantum efficiency (EQE) was characterized by measuring the incident photon-to-current conversion efficiency (IPCE) using a solar cell QE/IPCE measurement system (Solar Cell Scan 100, Zolix, Beijing, China). The stability tests were performed according to the protocols for OSCs [34]. Accordingly, the fabricated OSCs, which were encapsulated using the PUA resin (NOA63, Norland Optical Adhesive, Cranbury, NJ, USA), were maintained in the chamber (Daedong High Tech., Gimpo, Korea) at room temperature for ambient condition ~25 °C.

## 3. Results

**Figure 1:**
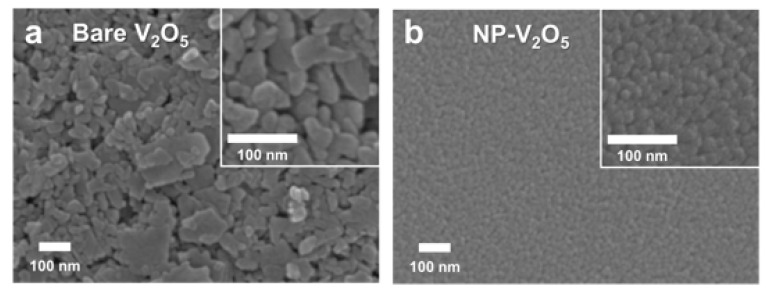
(**a**) Field-emission scanning electron microscopy (SEM) images of bare V_2_O_5_ on indium tin oxide (ITO) glass; (**b**) SEM images of nanoparticulate compact V_2_O_5_ (NP-V_2_O_5_) on ITO glass prepared by using 40 mM polyethylene glycol (PEG).

The SEM images show the surface morphology of the V_2_O_5_ layers deposited on the ITO glass (Figure 1). The bare V_2_O_5_ film prepared by using the precursor solution without the use of the additive, i.e., PEG, was composed of randomly agglomerated particles of which diameter is from a few tens to several hundreds of nanometers. The randomly agglomerated particles resulted in the microporous morphology on the V_2_O_5_ layer. However, the compact nanoparticulated morphology was obtained using the metal-organic decomposition method. The compact V_2_O_5_ film with globular nanoparticles was achieved using the precursor solution with 40 mM PEG (Figure 1c). We found the optimal condition by characterizing the SEM images of NP-V_2_O_5_ films prepared by various concentration of PEG (10~80 mM) in Figure S1, supporting information. The thickness of the V_2_O_5_ films was estimated by characterizing the cross-section SEM images of bare V_2_O_5_ (80–170 nm) and compact V_2_O_5_ prepared with 40 mM PEG (~80 nm) in Figure S2, Supplementary Information.

**Figure 2:**
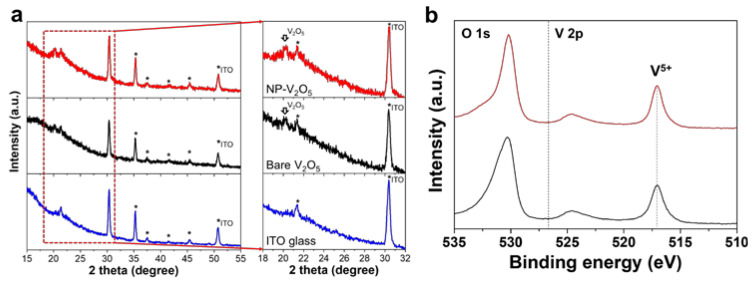
(**a**) X-ray diffraction (XRD) patterns of bare ITO glass, bare V_2_O_5_ on ITO glass, and NP-V_2_O_5_ on ITO glass prepared using 40 mM PEG. The peaks denoted by asterisk correspond to ITO glass; (**b**) V 2p XPS spectra of bare V_2_O_5_ on ITO glass, and NP-V_2_O_5_ on ITO glass prepared using 40 mM PEG.

XRD characterization was performed to investigate the crystallinity of the fabricated V_2_O_5_. As shown in Figure 2a, the peaks at ~20.3° assigned to the α-V_2_O_5_ phase (JCPDS card no. 89-0611) were observed in the bare V_2_O_5_ prepared using the precursor without the additive and the nanoparticulated compact V_2_O_5_ layer prepared using the precursor with 40 mM PEG [35,36,37]. The V 2p XPS spectrum was characterized to understand the surface oxidation state of the V_2_O_5_ films (Figure 2b) with the Al Kα X-ray. The strong peak at the binding energy of 517 eV, which was assigned to the V^5+^ of V_2_O_5_, was observed in both the bare V_2_O_5_ and the NP-V_2_O_5_ prepared using 40 mM PEG [38,39,40]. No significant changes in the XRD and XPS spectra were observed between the bare V_2_O_5_ and NP-V_2_O_5_ layers. This indicates that the crystalline structure and oxidation state were not affected by the use of the additive; however, the nanostructure morphology and surface uniformity of the V_2_O_5_ layers were affected by the additive.

**Figure 3:**
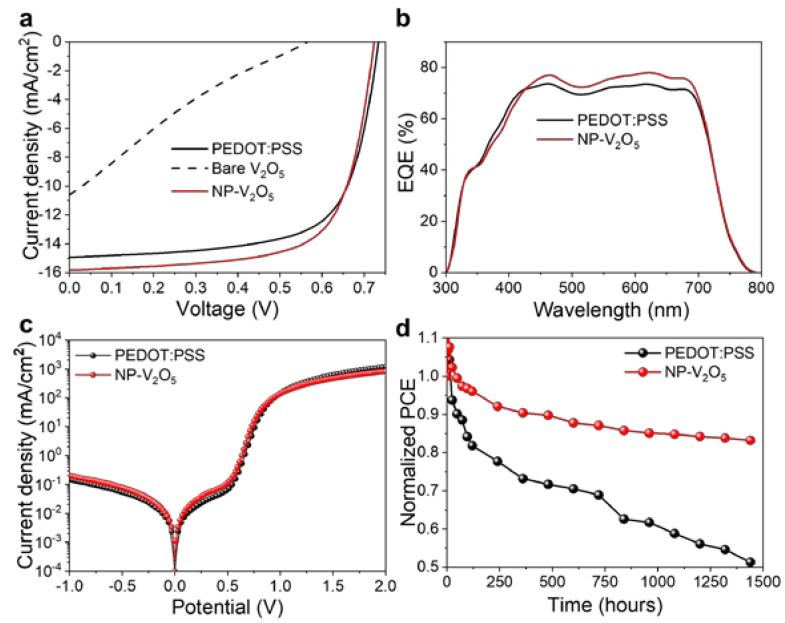
(**a**) Photocurrent density–voltage curves; (**b**) external quantum efficiency (EQE) curves; (**c**) logarithmic current density–voltage curves characterized under a dark condition; (**d**) normalized PCE values as a function of storage time for the OSCs. Here, PEDOT:PSS is poly(3,4-ethylenedioxythiophene):poly(styrenesulfonate).

Figure 3a and Figure S3, Supplementary Information, show the current density–voltage (*J–V*) curves of the OSCs with various HTLs under an illumination of 1 sun. The device performance parameters are summarized in Table S1, Supplementary Information. As a reference device, a PEDOT:PSS (AI 4083) layer with the thickness of approximately 40 nm was spin-coated on the surface of ITO glass, whose PCE, *V_oc_, J_sc_*, and *FF* values were 7.41%, 0.734 V, 14.95 mA/cm^2^, and 68.09%, respectively (Figure 3a). In contrast, a very low PCE value of 1.24% was obtained for the OSC with the bare V_2_O_5_ HTL prepared using the pristine precursor without the additive. For the reproducibility test, the *J–V* curves of 10 devices were characterized; however, 3 of them were not functioning. This is presumably due to the rough surface morphology and numerous pinholes on the bare V_2_O_5_ film. When the additive (i.e., PEG 300) was introduced in the precursor of the V_2_O_5_ HTL, the PCE value was significantly improved to over 7.1% (Figure S3, Supplementary Information). Due to the modified surface morphology and compactly packed nanoparticles, a slightly higher PCE value of 7.89% was obtained for the OSC with the NP-V_2_O_5_ HTL prepared using 40 mM PEG (Figure 3a). The *V_oc_, J_sc_*, and *FF* values of the OSC with NP-V_2_O_5_ were 0.723 V, 15.81 mA/cm^2^, and 69.01%, respectively. The EQE spectra of the OSCs were characterized by measuring the IPCE as well, and a significantly enhanced EQE of the OSC with NP-V_2_O_5_ can be observed in Figure 3b. The enhancement of EQE was well matched with the improvement of *J_sc_* values. The evaluated current density values from EQE were 14.1 mA/cm^2^ and 15.1 mA/cm^2^, respectively, for the OSCs with PEDOT:PSS and NP-V_2_O_5_. The logarithmic current density–voltage curves under a dark condition are shown in Figure 3c to investigate the electrical properties. The evaluated series and shunt resistance values were, respectively, 1.25 Ω m^2^ and 18.4 kΩ cm^2^ for the OSC with PEDOT:PSS, and 1.01 Ω m^2^ and 23.4 kΩ cm^2^ for the OSC with NP-V_2_O_5._ The series resistance value was slightly decreased; however, an approximately two-fold higher shunt resistance value was obtained. Since the shunt resistance parameter is strongly associated with the leakage current property of multi-layered devices in the horizonal direction. Thus, the significant increase in the shunt resistance indicates that the OSC with NP-V_2_O_5_ has significantly less leakage current than the OSC with PEDOT:PSS [41,42]. The decay of the PCE was characterized over 1440 h (60 days) according to the protocols for examining the stability of OSCs [34]. Figure 3d shows the stability of the OSCs stored in a chamber at the ambient temperature under the dark condition. As the strong acidic property of PEDOT:PSS dissolves the heavy-metal components in the ITO glass and deteriorates the stability of the OSCs, the incorporation of the NP-V_2_O_5_ film in the OSC, replacing PEDOT:PSS successfully, suppressed the rapid degradation of its initial performance [11]. The OSC with NP-V_2_O_5_ maintained 82% of its initial PCE value over 1440 h, whereas the OSC with PEDOT:PSS maintained 51% of its initial PCE value.

**Figure 4:**
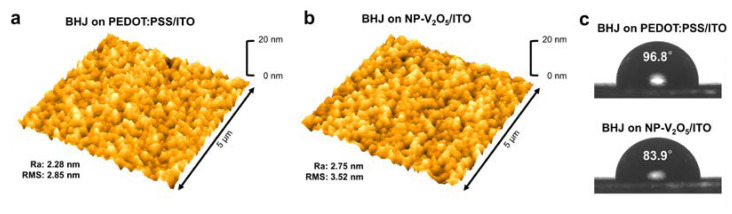
Atomic force microscopy (AFM) images of the bulk-heterojunction (BHJ) layer coated on the surface of (**a**) PEDOT:PSS and (**b**) NP-V_2_O_5_ films; (**c**) Contact angle images of water droplets (12 µL) on the surfaces of the BHJ/PEDOT:PSS and BHJ/NP-V_2_O_5_ films.

The *V_oc_* value of the OSC with NP-V_2_O_5_ was remarkably lower that that (0.734 V) of the OSC with PEDOT:PSS. As the *V_oc_* of OSCs strongly depends on the interfacial properties between the HTL and the polymer BHJ layer, the inorganic surface of V_2_O_5_ can possess an interfacial mismatch with the active layer, inducing some negative effects on the contact property. Moreover, the slightly higher work-function value of V_2_O_5_ (~4.7 eV) compared with that of PEDOT:PSS (~5.1 eV) can contribute to the lower *V_oc_* performance of the OSC with NP-V_2_O_5_ [21,22,23,24]. However, the modified surface energy of the HTL can promote the formation of the BHJ layer with a more favorable inner morphological structure, where the p-type donor polymer is enriched near the HTL and the n-type electron acceptor is enriched near the ETL [11]. This inner structure in the BHJ can strongly affect the charge separation and transport properties of the OSC, which are associated with the *J_sc_* and *FF* parameters. As the surface energy of the donor polymer (PTB7) is much lower than that of the electron acceptor (PC_70_BM), the noticeably reduced surface energy of the NP-V_2_O_5_ layer facilitates a more favorable vertical configuration in the BHJ layer [11,43]. The changes in the surface energy of the HTL were estimated via the contact angle measurements (Figure S4, Supplementary Information). Notably, no significant changes were observed in the surface roughness of the BHJ layers coated on the PEDOT:PSS and NP-V_2_O_5_ layers (Figure 4a,b). The root mean square (RMS) values were 2.28 nm for the BHJ layer coated on PEDOT:PSS/ITO and 2.75 nm for the BHJ layer coated on NP-V_2_O_5_/ITO. The slight increase in RMS value might be resulted from the relatively rough surface morphology of NP-V_2_O_5_ film (2.63 nm) comparting to that of PEDOT:PSS film (1.98 nm). However, the contact angle of a water droplet on the surface of the BHJ was reduced from 96.8° for the BHJ coated on PEDOT:PSS to 83.9° for the BHJ coated on NP-V_2_O_5_ (Figure 4c). This indicates that the electron acceptor was relatively enriched near the surface of the BHJ layer and the donor polymer was relatively enriched near the bottom of the BHJ layer when NP-V_2_O_5_ was introduced to the HTL. Therefore, the enhanced *J_sc_* and *FF* parameters of the OSC with NP-V_2_O_5_ are attributed to the modified inner morphological structure of the BHJ layer.

## 4. Conclusions

A solution-processable compact vanadium oxide (V_2_O_5_) film with a globular nanoparticulate structure was introduced to the HTL of polymer-BHJ-based solar cells by using a facile metal–organic decomposition method. Polyethylene glycol (PEG, *M_n_* 300) was used as the structure-determining surfactant in the precursor solution to fabricate the nanoparticulate compact V_2_O_5_ with a smooth surface morphology. The introduction of the nanoparticulate compact V_2_O_5_ HTL via the solution process with a neutral pH condition successfully improved the stability over 1440 h (60 days) by preventing the decomposition of the ITO glass, and the penetration of heavy-metal components and moisture. Moreover, the modified surface energy and improved surface properties of NP-V_2_O_5_ resulted in a slightly enhanced PCE value from 7.47% to 7.89% with a significant improvement in the short-circuit current density (*J_sc_*), and fill factor (*FF*). Thus, introducing a nanoparticulate crystalline metal oxide film with a compact morphology can be a promising strategy to replace the conventionally utilized PEDOT:PSS for diverse organic optoelectronics applications with enhanced long-term stability.

## Supplementary Materials

The following are available online at https://www.mdpi.com/2073-4360/12/8/1791/s1, Figure S1. SEM images of NP-V2O5 films prepared by using various concentrations of PEG (a) 10 mM, (b) 20 mM, and (c) 80 mM. Figure S2. Cross-section SEM images of (a) bare V2O5 and (b) NP-V2O5 prepared using 40 mM PEG. Figure S3. Photocurrent density–voltage curves of the OSCs with NP-V2O5 HTL prepared using various concentrations of PEG from 10 mM to 80 mM. Figure S4. Contact angle and AFM images of water droplets (12 μL) on the surface of (a) PEDOT:PSS and (b) NP-V2O5 films prepared using 40 mM PEG. Table S1. Summary of the device performance parameters.
