# Supplementary material for: Metal-Organic Decomposition-Mediated Nanoparticulate Vanadium Oxide Hole Transporting Buffer Layer for Polymer Bulk-Heterojunction Solar Cells
Source: Polymers (Basel). 2020 Aug 10;12(8):1791. doi: 10.3390/polym12081791 (PMC7465065; doi:10.3390/polym12081791)
Supplement: Supplementary file 1 [file polymers-12-01791-s001.pdf]

Supplementary Information

Metal-Organic Decomposition-Mediated Nanoparticulate Vanadium Oxide Hole Transporting Buffer Layer for Polymer Bulk-heterojunction Solar Cells

Chengkai Xia, Won Tae Hong, Young Eun Kim, Woo-Seok Choe, Dong-Hwan Kim * and Jung Kyu Kim *

School of Chemical Engineering, Sungkyunkwan University (SKKU), Suwon 16419, Korea

* Correspondence: dhkim1@skku.edu (D.H.K.); legkim@skku.edu (J.K.K.); Tel.: +82-31-299-4717 (D.H.K.); +82-31-290-7254 (J.K.K.)

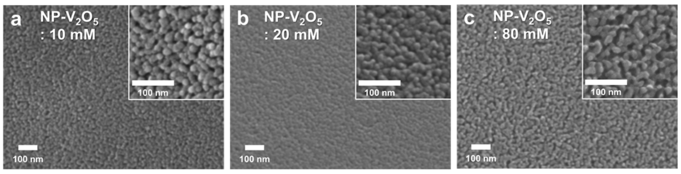

Figure S1. SEM images of NP-V_2O_5 films prepared by using various concentrations of PEG (a) 10 mM, (b) 20 mM, and (c) 80 mM.

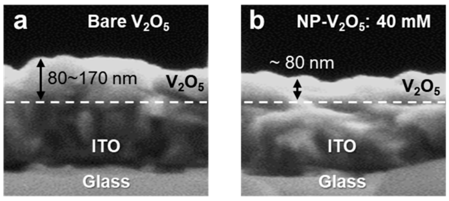

Figure S2. Cross-section SEM images of (a) bare V_2O_5 and (b) NP-V_2O_5 prepared using 40 mM PEG.

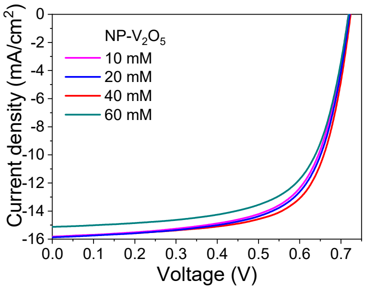

Figure S3. Photocurrent density–voltage curves of the OSCs with NP-V_2O_5 HTL prepared using various concentrations of PEG from 10 mM to 80 mM.

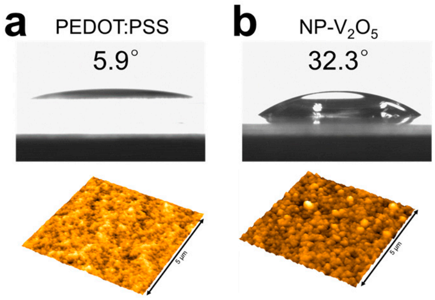

Figure S4. Contact angle and AFM images of water droplets (12 μL) on the surface of (a) PEDOT:PSS and (b) NP-V_2O_5 films prepared using 40 mM PEG.

Table S1. Summary of the device performance parameters.

Devices [a,b]	V_{oc} (V)	J_{sc} (mA/cm^2)	FF (%)	PCE (%)
PEDOT:PSS	0.734 (±0.01)	14.95 (±0.02)	68.09 (±0.11)	7.47 (±0.09)
Bare V_2O_5	0.562 (±0.54)	10.06 (±4.58)	20.70 (±9.24)	1.24 (±1.09)
NP-V_2O_5 (10 mM PEG)	0.725 (±0.07)	15.79 (±0.04)	66.47 (±0.24)	7.55 (±0.22)
NP-V_2O_5 (20 mM PEG)	0.723 (±0.04)	15.84 (±0.02)	67.12 (±0.18)	7.68 (±0.19)
NP-V_2O_5 (40 mM PEG)	0.723 (±0.05)	15.81 (±0.03)	69.01 (±0.15)	7.89 (±0.11)
NP-V_2O_5 (80 mM PEG)	0.717 (±0.13)	15.10 (±0.37)	66.04 (±0.41)	7.15 (±0.52)

[a] The cell area was determined by using an aperture of area 11.43 mm^2. The aperture was placed on top of the cell (of approximately 15 mm^2). These area values were carefully characterized with a video microscope (Sometech, SV-35). [b] The average values were obtained from 10 devices for each condition.
